# Cost-utility analysis of a preventive home visit program for older adults in Germany

**DOI:** 10.1186/s12913-015-0817-0

**Published:** 2015-04-03

**Authors:** Christian Brettschneider, Tobias Luck, Steffen Fleischer, Gudrun Roling, Katrin Beutner, Melanie Luppa, Johann Behrens, Steffi G Riedel-Heller, Hans-Helmut König

**Affiliations:** Department of Health Economics and Health Services Research, University Medical Center Hamburg-Eppendorf, Martinistrasse 52, D-20246 Hamburg, Germany; University of Leipzig, Institute of Social Medicine, Occupational Health and Public Health, Leipzig, Germany; University of Leipzig, LIFE – Leipzig Research Center for Civilization Diseases, Leipzig, Germany; Martin-Luther-University Halle-Wittenberg, Institute for Health and Nursing Science, Halle, Germany; Private University of Witten/ Herdecke, Institute for Integrative Medicine (IfIM), Integrated Curriculum for Anthroposophic Medicine (ICURAM), Witten, Germany; German Institute for Economic Research DIW, Berlin, Germany; ISIS-Institute for Supervision-, Institutions- and Social Research, Frankfurt a. M, Germany

**Keywords:** Cost benefit analysis, Home care services, Community medicine, Geriatrics, Independent living, Aged, 80 years and over, Quality-adjusted life years

## Abstract

**Background:**

Most older adults want to live independently in a familiar environment instead of moving to a nursing home. Preventive home visits based on multidimensional geriatric assessment can be one strategy to support this preference and might additionally reduce health care costs, due to the avoidance of costly nursing home admissions. The purpose of this study was to analyse the cost-effectiveness of preventive home visits from a societal perspective in Germany.

**Methods:**

This study is part of a multi-centre, non-blinded, randomised controlled trial aiming at the reduction of nursing home admissions. Participants were older than 80 years and living at home. Up to three home visits were conducted to identify self-care deficits and risk factors, to present recommendations and to implement solutions. The control group received usual care. A cost-utility analysis using quality-adjusted life years (QALY) based on the EQ-5D was performed. Resource utilization was assessed by means of the interview version of a patient questionnaire. A cost-effectiveness acceptability curve controlled for prognostic variables was constructed and a sensitivity analysis to control for the influence of the mode of QALY calculation was performed.

**Results:**

278 individuals (intervention group: 133; control group: 145) were included in the analysis. During 18 months follow-up mean adjusted total cost (mean: +4,401 EUR; bootstrapped standard error: 3,019.61 EUR) and number of QALY (mean: 0.0061 QALY; bootstrapped standard error: 0.0388 QALY) were higher in the intervention group, but differences were not significant. For preventive home visits the probability of an incremental cost-effectiveness ratio <50,000 EUR per QALY was only 15%. The results were robust with respect to the mode of QALY calculation.

**Conclusions:**

The evaluated preventive home visits programme is unlikely to be cost-effective.

**Trial registration:**

Clinical Trials.gov Identifier: NCT00644826.

## Background

Due to demographic change the group of older adults in Germany and most other European countries will increase substantially within the coming decades [1]. Most older adults prefer to grow old in the community within a familiar environment instead of moving to a nursing home. Preventive home visits based on multidimensional geriatric assessment can be one measure to support this preference and might additionally reduce health care costs, due to the avoidance of costly nursing home admissions. However, compared to single interventions in community-dwelling populations like fall prevention programs [2,3], the effectiveness of preventive home visits remains uncertain [4-8]. In their systematic review Huss et al. concluded that multidimensional preventive home visits have the potential to reduce disability burden [8]. However, effects on nursing home admissions were heterogeneous. Huss et al. explained these variations between studies by means of four major factors: intervention program characteristics, population characteristics, adherence to recommendations made by the program and the specific setting [8]. Besides the vast literature on the effectiveness, there are only a few studies on the cost-effectiveness of preventive home visits, using different outcome measures like active life-years gained or successful treatment [9-13], but neglecting the effects on health-related quality of life. Furthermore, to our knowledge no study has assessed resource use associated with informal care which - from a societal perspective – is a major cost category in populations of older aduts [14].

To measure the impact of preventive home visits on health-related quality of life and to enable comparison with other (preventive) interventions for other conditions, cost-utility analyses can be conducted. Internationally, only one cost-utility analysis of preventive home visits in Sweden has been conducted so far [11]. However, health-related quality of life was only measured post hoc.

The purpose of this study was to perform a cost-utility analysis of preventive home visits from a societal perspective in Germany.

## Methods

### Sample

This study is part of a 18-month multi-centre, non-blinded, randomised controlled trial focusing on the effectiveness of preventive home visits in terms of reduction of nursing home admissions (primary endpoint) (Clinical Trials.gov Identifier: NCT00644826). The methods have been described in detail elsewhere [15,16]. In brief, study participants were recruited in two regions of Eastern Germany (Halle and Leipzig) via GP practices (in both regions), hospitals and the registration offices (only in Halle). Individuals were recruited between August 2007 and July 2008. Participants had to be older than 80 years, residents of Leipzig or Halle, and had to live at home or – in case of hospital patients – discharge to home had to be planned already. Individuals were excluded if they had insufficient German language skills, suffered from cognitive impairment, were not able to give informed consent or had a care level >1 (according to German long term care insurance [17]). This means that patients were excluded if they needed assistance in more than two activities of basic nursing (e.g. personal hygiene, feeding, mobility) more than once a day. To be eligible for care level 1 the maximum amount of care must not exceed 3 hours a day.

**Figure 1:**
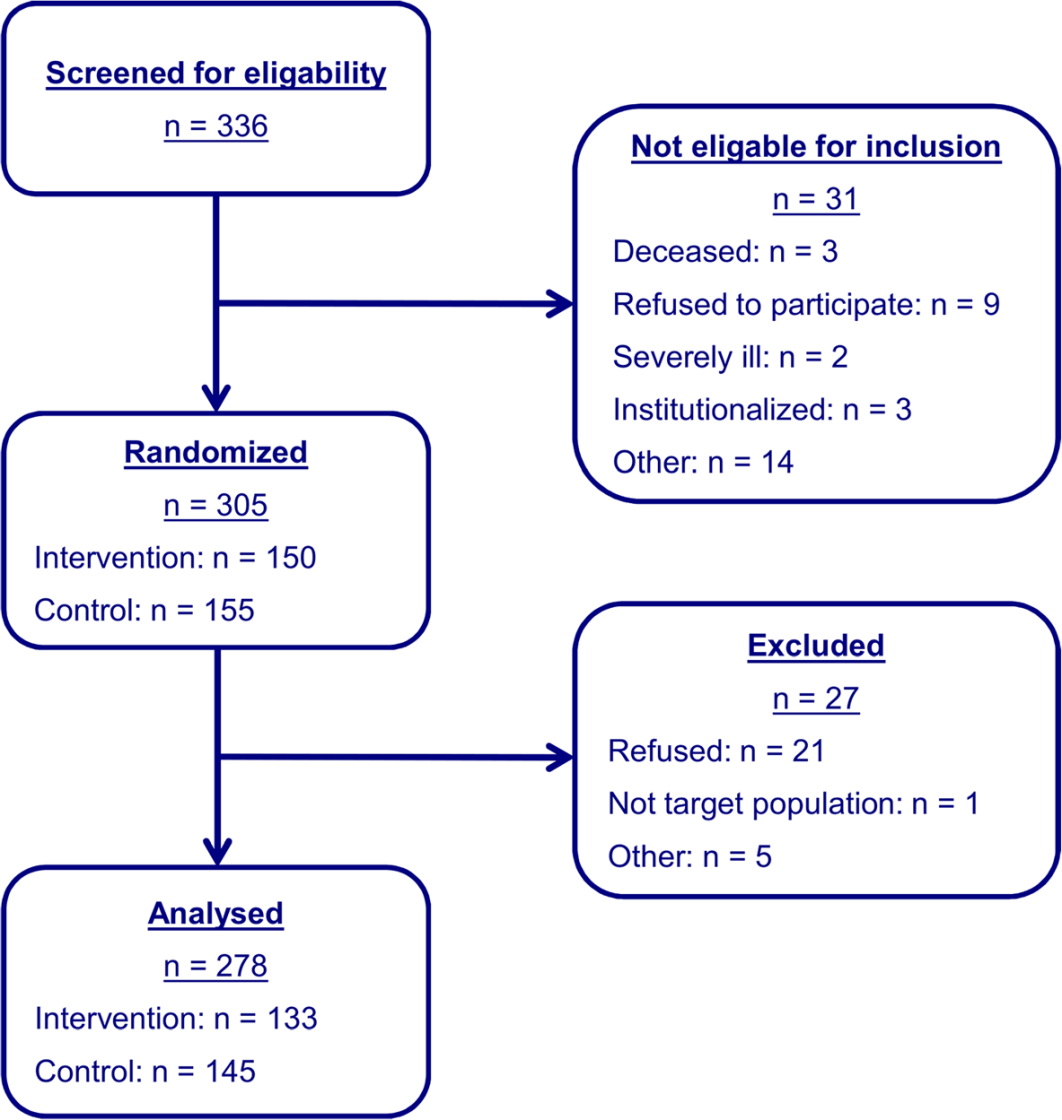
**Flow chart of patient inclusion and exclusion.**

Participants were randomised to intervention and control group using a balanced block-wise randomisation stratified by region. The sample size required to detect a reduction of nursing home admissions from 20% to 7% with 80% power at a significance level of 5% assuming a drop-out rate of 30% was calculated to be n = 320 [15]. In total n = 336 adults older than 80 years gave informed consent to participate in the study and were screened for eligibility. 31 participants were not eligible. 305 older adults were randomised. N = 150 received the intervention and n = 155 received usual care. One further patient of the IG was excluded from the analysis ex post. This patient was not part of the target population of the intervention as he received 24 hour professional care at home and was unable to live independent in his or her own home. Finally, n = 304 patients were part of this analysis (Figure 1). The time horizon of the RCT was 18 months.

### Intervention

In the intervention group (IG) a multidimensional geriatric assessment was performed by trained personnel (nursing scientist, psychologist or sociologist) in a first preventive home visit. In this first visit the current nutrition status, the impairment of sight and hearing, urinary and bowel incontinence and the loss of functional muscle mass was assessed. Furthermore social activities, housing conditions, economic conditions, polypharmacy and the cognitive status were determined.

A case conference was conducted by a multi-professional team (nursing scientist, psychologist, geronto-psychiatrist, nutritionist, social worker) within three weeks after the geriatric assessment to work out individualized recommendations based on an analysis of identified self-care deficits and risk factors for institutionalization.

After the case conference the participants of the IG were visited again for the second preventive home visit by the personnel who performed the first visit. The visitor reported to the participant on identified self-care deficits and risk factors and presented the results and recommendations of the case conference.

Four weeks after the second visit the third preventive home visit was arranged. In this visit, also called booster session, the adherence to the recommendations was evaluated. Furthermore, obstacles and facilitators of adherence were identified, the recommendations were reviewed, and further support was offered. The researchers were not involved in carrying out the intervention.

The control group (CG) received usual care (i.e. every service offered by the statutory health insurance system and utilized by the patient on his own initiative).

### Data collection and measures

#### Data collection

The data collection process has been described in detail elsewhere [15]. In brief, all study participants at baseline (2007/2008) and follow-up (18 months) were interviewed face-to-face by skilled and trained staff of the two study centres.

Besides the geriatric assessments, data on socio-demography (age, gender, living situation, and education), basic activities of daily living (Barthel Index [18]), instrumental activities of daily living (IADL by Lawton and Brody [19]), cognitive status (Mini Mental State Examination (MMSE) [20]), symptoms of depression (Geriatric Depression Scale (GDS) [21]) as well as health-related quality of life (EQ-5D), service use and patient costs were collected.

In a few cases where a face-to-face interview at follow-up was declined, telephone interviews with the respondent or proxy interviews (e.g. family member) were offered. In addition, if a respondent developed a cognitive impairment during follow-up proxy interviews were also conducted.

Drop-out rates were rather low, resulting in n = 133 (89%) participants analysed in the IG and n = 145 (94%) in the CG (Figure 1). Drop-outs were not significantly different from analysed participants with respect to the group they were allocated to (*χ*^*2*^-test, p-value = 0.188), study centre (*χ*^*2*^-test, p-value = 0.847), gender (*χ*^*2*^-test, p-value = 0.690), and age (t-test, p-value = 0.834). However, more older adults who were living alone (n = 21) did not continue the study (*χ*^*2*^-test, p-value = 0.046). These patients were not different from patients finishing the study in terms of baseline costs and quality of life.

#### EQ–5D-3 L

The EQ–5D-3 L is a generic health-related quality of life (HRQL) questionnaire that comprises five questions (items) relating to current problems in the domains: mobility; self-care; usual activities; pain/discomfort; and anxiety/depression [22]. Responses in each dimension are divided into three ordinal levels coded: 1, no problems; 2, moderate problems; 3, extreme problems. Theoretically, 3^5^ = 243 different health states can be defined by the EQ–5D descriptive system.

The EQ–5D also includes a visual analogue scale (EQ–VAS), similar to a thermometer, ranging from 0 (worst imaginable health state) to 100 (best imaginable health state) which records the respondent’s self-rated valuation of health state (EQ–VAS score).

The EQ-5D has been used successfully in the populations of older adults [23]. Furthermore, according to a particular set of societal preference values derived from surveys of the general population, an index score (EQ–5D index) for each of the 243 EQ–5D health states is available for various countries, with the best state (perfect health) and ‘death’ being assigned values of one and zero, respectively. In the present study EQ–5D index scores from the UK [24] were used that were derived from a large general population sample (n = 2,997). The existing German value set was not used as it does not consider problems in usual activities and hence would result in imprecise values [25]. Accordingly, to each participant’s health status on the descriptive system of the EQ–5D, an EQ–5D index score was assigned.

#### Questionnaire of service utilization and costs

Service use and patient costs for baseline and follow-up were measured from a societal perspective. The questionnaire was based on service use questionnaires used in earlier studies [26-29], which were adapted to the purposes of the present study, and the German adaptation of the Resource Utilisation in Dementia (RUD) questionnaire [30] to assess informal care. Informal care was assessed by two questions focussing on time spent on assistance in basic activities of daily living (e.g. toilet use, dressing, bathing) and in instrumental activities of daily living (e.g. cooking, support in financial issues, shopping, housekeeping).

To minimize recall bias resource utilisation was assessed retrospectively over different time periods. Formal and informal nursing care, outpatient physician services, pharmaceuticals, use of outpatient non-physician services (e.g. occupational therapy, physiotherapy, logopedics, sports therapy) medical supply and dentures as well as transportation to and from medical treatments were assessed over 3 months. Inpatient services were recorded during the last 12 months and costs for in-home modifications during the past 18 months. Depending on the service, quantity of use or duration was recorded (Table 1). To further account for recall bias, lists of possible services were presented (e.g., every outpatient service was addressed specifically in the questionnaire). Indirect costs due to productivity losses with respect to paid and unpaid work and reduced productivity were not included, since they are of minor importance in this older population.

**Table 1:** **Resources and unit costs used for cost calculation**

**Sector**	**Service/goods**		**Units**	**Monetary values (unit costs)**
Nursing care	Formal care	Nursing home care	Days	Type specific mean rates by level of care, market prices [32]
		Ambulatory care	Hours	Type specific wage [33,34]
	Informal care		Hours	Type specific wage (replacement cost approach) [33,34]
Inpatient services	Acute hospital and hospitals for rehabilitation		Days	Type specific mean rates [35-37]
Medication	Product		Quantity	Official pharmaceutical index (Rote Liste) [39]
Outpatient physician services	GP, specialists (e.g. cardiologist, internist, ophthalmologist)		Contacts	Type specific mean rates [38]
Outpatient non-physician services	e.g. physiotherapy, massage, lymph drainage, ergotherapy		Contacts	Reimbursement schedule [40-42]
Medical devices and dentures	Product		Quantity	Market prices and reimbursement schedules [38,43,44]
Transportation	Transportation by car, public transport, taxi or ambulance		km, quantity	Market prices, EUR 0.30 per km for transport by car [45]

GP = General practitioner; km = kilometer.

#### Unit costs

To determine direct health care costs, unit costs were calculated for all services used and for all goods privately purchased or prescribed. Costs were determined for the different time periods of resource utilisation and then extrapolated to 18 months by multiplying them by 6 (in case of 3-month assessment) or 1.5 (in case of 12-month assessment), respectively. Costs were calculated in EUR at 2008 price level. If unit cost data was only available for years before 2008, costs were inflated using the consumer price index [31]. Costs were not discounted because of the short time horizon of 18 month.

Detailed information regarding monetary valuation is shown in Table 1. In brief, nursing home care was valued using calculated costs of care per day, differentiated by the level of care [32] and market prices for assisted living. Professional nursing care was valued using the average hourly gross wage rate plus non-wage labour costs for employees in the domain of care and assistance for the older adults or handicapped (18.69 EUR per hour) [33,34]. Informal care was valued using the replacement cost method, i.e. it was assumed that informal care could have been substituted by paying a professional caregiver, and hours of informal care were therefore valued using the same hourly wage rate as for the valuation of professional nursing care. For the valuation of any in-home modifications the self-reported costs were used.

Inpatient hospital services were valued using average costs per diem differentiated by the type of the hospital [35-37]. Costs of outpatient physician services were calculated based average costs per contact [38]. Pharmaceuticals were valued based on drug codes, dosage and duration as recorded in the questionnaire in conjunction with a major German pharmaceuticals database [39]. Outpatient non-physician services were valued using reimbursement schemes of the German statutory sickness funds [40-42]. Costs for medical supply and dentures were calculated using market prices according to patients’ specifications. If no specification was available reimbursement schemes were used [38,43,44]. Costs for travel on public transport or by taxi were calculated according to patients’ specifications. Costs of car travel were calculated according to the number of kilometres travelled multiplied by a flat rate of 0.30 EUR per kilometre (according to the tax-deductible rate allowed for trips to and from work in Germany, [45]).

#### Intervention costs

To calculate total costs, intervention costs were added in the IG to direct health care costs. Intervention costs consisted of costs for the geriatric assessments, the case conferences and the following home visits. Furthermore travel costs to the participants were considered. Cost calculations were based on recorded staff time and the number of staff taking part in the case conferences. Staff time was valued with an average hourly gross wage rate plus non-wage labour costs for employees as done for professional nursing services [46,47]. It was assumed that overhead costs were low because, for example, no additional room had to be rented and available meeting rooms were not fully occupied with respect to time.

### Data analysis

#### Calculation of costs

Unadjusted individual total costs C_i_ at follow-up were calculated for each participant i. The follow-up period started with the first home visit. Total costs for each individual were estimated by summing the 18-month costs of all costs categories as described above. To take into account the individual observation time the following equation for each individual i was used:

1 $$ {C}_i=\frac{C_{T1}}{548 days}\times days\left(T0;T1\right)\Big) $$

where C_T1_ are the individual total costs measured at T1 over 18 months (548 days), and days (T0;T1) are the number of days between the measurement points for the individual patient.

Participants were excluded, if no information about costs was retrievable at T1. If the participant had died during follow-up the cost value for T1 was replaced by the value of T0 which was adjusted by the individual observation time. Costs were not discounted.

#### Calculation of QALY

QALY were calculated by weighting the duration of health states by the EQ–5D index. Individual QALY_i_ at follow-up were calculated for each participant i using linear interpolation between measurement points and taking into account the individual observation time, using the following equation:

2 $$ QAL{Y}_i=\left[\frac{inde{x_T}_0+ inde{x}_{T1}}{2}\times days\left(T0;T1\right)\right]/365 $$

where index_T0_ and index_T1_ are the individual EQ–5D index scores measured at T0 and T1. The procedure to treat deaths described above for costs was also used for the computation of QALY: The EQ-5D index for T1 was replaced by the EQ-5D index for T0 which was multiplied by the duration of study participation. QALY were not discounted.

#### Statistical analysis

Missing data were rare (0.1% of health economic items at baseline; 0.3% of health economic items at follow-up) and were replaced by the unconditional mean of the respective variable. The level of significance was set at 5%. Differences in means and proportions of baseline sample characteristics were analysed using t-test with bootstrapped standard errors (4,000 replications) and χ^2^-test, respectively. Differences in mean costs and mean QALYs between IG and CG were adjusted for study region, age and gender as well as for baseline HRQL, 12-month costs at baseline, cognition, basic and instrumental quality of life and depressive symptoms by means of OLS regression with bootstrapped standard errors.

Net monetary benefit regression with different willingness to pay thresholds was used to construct cost-effectiveness acceptability curves (CEAC) controlled for potential prognostic variables [48]. The individual net monetary benefit (NMB_i_) was calculated for each participant i as:

3 $$ NM{B}_i= QALYi\times WTP- Ci $$

were QALY_i_ and C_i_ are the effects and cost for each participant i, respectively, and WTP is the willingness to pay for a QALY. We used an ordinary least square (OLS) regression with bootstrapped standard errors (4,000 replications) and controlled for study region, age and gender as well as for baseline HRQL, 12-month costs at baseline, cognition, basic and instrumental quality of life and depressive symptoms.

All statistical analyses were carried out on an intention-to-treat basis using the software package STATA for Windows, Release 13 (STATA Corp., College Station, Texas, USA) as well as PASW Statistics 18.0.0 (SPSS Inc., Chicago, IL).

#### Sensitivity analysis

As stated above we performed a sensitivity analysis based on the complete sample of 279 patients. Furthermore, to analyse the sensitivity of the results with respect to the effect measure, univariate sensitivity analyses were performed. QALY were calculated based on the EQ–5D index scores derived from a much smaller sample of the German general population (n = 334) [25] and, alternatively, EQ–VAS scores (divided by 100 for transformation to a 0–1 scale) were used as QALY weights.

### Ethics

The study was approved by the ethics committees of the universities Halle and Leipzig. Written informed consent was obtained. All clinical investigations have been conducted according to the principles expressed in the Declaration of Helsinki. No allowance for the participation in the study was paid.

## Results

### Characteristics of the study population at baseline

The characteristics of the study population are presented in Table 2. There were no significant differences in the baseline characteristics between the IG and the CG. Respondents had a mean age of 85 years, more women than men (IG 66%, CG 72%) participated in this study and the majority of respondents had no level of care in the long-term care insurance (IG 82%, CG 73%). The mean EQ-5D index at baseline was not significantly different (IG: 0.59; CG: 0.60), resulting in mean numbers of QALYs over 18 months at baseline of 0.8907 in the IG and 0.8954 in the CG, respectively. There was no statistically significant difference in mean 18-months costs (IG: 16,568 EUR; CG: 18,451 EUR).

**Table 2:** **Comparison of sample characteristics at baseline (n = 304)**

**Characteristics**	**Intervention group (n = 149)**	**Control group (n = 155)**	**p-value**
Age (years)			0.72*
mean (SD)	84.85 (3.54)	84.70 (3.47)	
Female: n (%)	99 (66.44)	111 (71.61)	0.33**
Living situation : n (%)			0.72**
Alone	94 (63.09)	105 (67.74)	
With spouse/partner	43 (28.86)	43 (27.74)	
With relatives	8 (5.37)	6 (3.87)	
Other	3 (2.01)	1 (0.65)	
Training qualification: n (%)			0.67**
None	29 (19.46)	34 (21.94)	
Vocational training	93 (62.42)	89 (57.42)	
University	27 (18.12)	32 (20.65)	
Level of care dependency: n (%)			0.16**
None	122 (81.88)	113 (72.90)	
Level I	25 (16.78)	38 (24.52)	
Level II	2 (1.34)	4 (2.58)	
Level III	0 (0.00)	0 (0.00)	
EQ-5D index			0.92*
mean (SD)	0.59 (0.28)	0.60 (0.28)	
EQ VAS			0.64*
mean (SD)	58.41 (19.27)	59.36 (16.50)	
18-month costs (€; 2008)			0.49*
mean (SD)	16,568 (22,221)	18,451 (25,685)	

*t-test (p-values based on non-parametric bootstrapping (4000 replications)); **Chi^2^-test; SD: Standard deviation.

### Nursing home admissions

As has been reported elsewhere [49] the number of nursing home admissions (primary endpoint of the trial) was smaller in the intervention group (n = 8) than in the control group (n = 15), resulting in an adjusted hazard ratio (HR) of 0.55 which means that the risk of admission to nursing home was reduced by 45%. However, this result was not statistically significant (95%-CI of HR: 0.23 - 1.30). Thus, although the number of nursing home admissions was reduced the intervention did not reach its primary goal.

### Costs during follow-up

Mean total unadjusted costs in the IG (20,195 EUR) were 833 EUR lower than in the CG (21,028 EUR) (Table 3). Yet, total adjusted costs were 4,440 EUR higher in the IG than in the CG (p = 0.15) (Table 4). In both groups the most important cost drivers were informal care (unadjusted 8,802 EUR in IG; 7,434 EUR in CG) and inpatient hospital care (unadjusted 3,479 EUR in IG; 3,809 EUR in CG) (Table 3). The high costs of informal care were mainly caused by assistance in instrumental activities of daily living (unadjusted 5,951 EUR in IG; 5,273 EUR in CG). Based on adjusted costs there were only two statistically significant differences in the cost categories between IG and CG: The intervention was associated with higher costs for informal care (+4,968 EUR; p =0.03) and for outpatient physician services (+406 EUR; p = 0.03) (Table 4). Adjusted costs for nursing home care were not significantly different between IG and CG.

**Table 3:** **Unadjusted costs (by cost category) and QALY at 18-month follow-up**

**Cost category**	Intervention group (n = 133)*	Control group (n = 145)*
Total costs		
Mean (SD)	20,195 (21,689)	21,028 (24,384)
Intervention costs		
Mean (SD)	73 (22)	0 (0)
Inpatient services		
Mean (SD)	3,479 (8,183)	3,809 (8,603)
Outpatient services (physician)		
Mean (SD)	857 (1,198)	575 (531)
Outpatient services (non-physician)		
Mean (SD)	462 (823)	406 (1,323)
Medication		
Mean (SD)	1,385 (1,197)	1,426 (1,462)
Medical devices		
Mean (SD)	425 (992)	321 (1,137)
Nursing home care		
Mean (SD)	1,228 (5,833)	2,197 (8,519)
Ambulatory care (nursing service)		
Mean (SD)	2,228 (4,280)	1,987 (4,430)
Informal care		
Mean (SD)	8,802 (16,714)	7,434 (16,287)
Modification of buildings		
Mean (SD)	205 (1,142)	44 (331)
Transportation		
Mean (SD)	113 (163)	145 (262)
QALY		
Mean (SD)	0.8256 (0.4029)	0.8270 (0.4097)

*Differences from n = 149 and n = 155 owing to missing values, due to withdrawn consent and no data on service use retrievable; outpatient services (non-physician) contains: physiotherapy, occupational therapy, logopedics, sports therapy, massage, thermal therapy).

**Table 4:** Adjusted* differences intervention group and control group in mean costs (by cost category) and QALY (n = 278)

	**Difference (Intervention – control)**	**Bootstrapped standard error**	**p value**
Total cost [€]	4,400.52	3,019.61	0.15
Inpatient services [€]	944.82	1,088.66	0.39
Outpatient, physician services [€]	405.59	186.95	0.03**
Outpatient, non-physician services [€]	−7.16	230.32	0.98
Medication [€]	−60.03	220.42	0.79
Medical devices [€]	−159.15	207.07	0.44
Nursing home [€]	1.15	1,227.12	0.99
Ambulatory care [€]	−316.44	740.52	0.67
Informal care [€]	4,968.09	2,343.03	0.03**
Modification of buildings [€]	340.78	223.98	0.13
Transport [€]	9.01	27.45	0.74
QALY	0.0061	0.0388	0.88

*Adjusted for study region, age and gender as well as for baseline HRQL, 12-month costs at baseline, cognition, basic and instrumental abilities of daily living and depressive symptoms by OLS regression; **p ≤ 0.05.

### Survival, quality of life and QALY during follow-up

26 participants died in the CG, 12 died in the IG. However, deceased participants in the CG lived 331 days and deceased participants in the IG only 310 days on average. The mean survival time was 508 days in the IG and 492 days in the CG. The mean unadjusted EQ-5D index score decreased slightly from baseline to follow-up in both groups, reaching 0.5563 (SD: 0.3068) in the IG and 0.5503 (SD: 0.3165) in the CG. The mean unadjusted number of QALY during follow-up was slightly higher in the CG (IG: 0.8256 QALY; CG: 0.8270 QALY, Table 3). Yet, after adjustment the mean number of QALYs was slightly, but insignificantly higher in the IG (+0.0061 QALY; p = 0.88) (Table 4).

### Base case analysis

**Figure 2:**
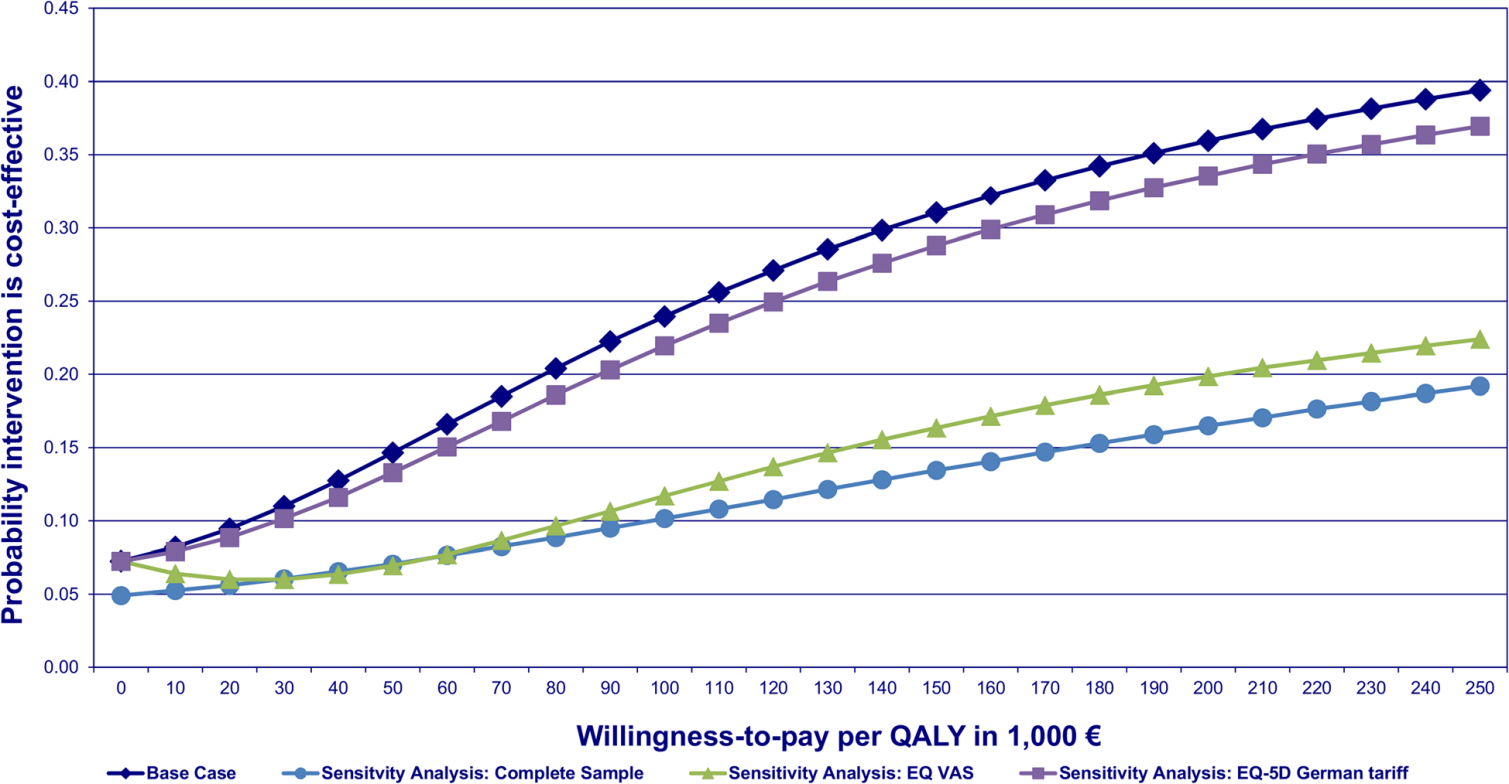
**Cost-effectiveness acceptability curves of the base case and sensitivity analyses.**

After controlling for prognostic variables via net monetary benefit regression as described above the probability of an ICER <50,000 EUR per QALY was only 15% for preventive home visits. The probability of cost-effectiveness increased with increasing WTP as shown by the CEAC (Figure 2). At a WTP of 0 EUR per QALY the probability of cost-effectiveness of preventive home visits was 7%, while at a WTP of 250,000 EUR per QALY the probability was 39%. We identified four variables that had a significant effect on the NMB over a wide range of WTP. Higher quality of life at baseline, less baseline limitations in instrumental activities of daily life and less depressive symptoms led to a higher NMB. On the other hand higher costs at baseline resulted in a lower NMB. Yet, this effect was just marginal and not stable under the influence of increasing WTP. The results of the net-benefit regression at a WTP threshold of 50,000 per QALY are shown in Table 5.

**Table 5:** **Net monetary benefit regression with WTP set at 50,000 € per QALY**

**Variable**	**Coefficient**	**p value**
	**b (SE)**	
Group (ref.: control group)	−4,096.59 (3,894.24)	0.29
Study center (ref.: Leipzig)	−3,711.82 (5,016.88)	0.46
Group*Study Center	8,446.42 (5,904.94)	0.15
Age at baseline (centered)	623.33 (422.18)	0.14
Gender (ref.: male)	3,888.47 (3,732.30)	0.30
EQ-5D index at baseline (centered)	41,391.13 (7,215.78)	0.00**
18-month costs at baseline (centered)	−0.44 (0.22)	0.04*
MMSE score at baseline (centered)	922.17 (721.08)	0.20
Barthel index score at baseline (centered)	221.85 (206.14)	0.28
IADL score at baseline (centered)	3,570.256 (1,534.46)	0.02*
GDS score at baseline (reversed, centered)	3,529.91 (1,759.86)	0.05*
Intercept	19,761.49 (4,284.06)	0.00

n = 278; bootstrapped standard errors (SE) based on 4,000 replications; R^2^: 0.55; GDS: Geriatric depression scale; IADL: Instrumental activities of daily living; MMSE: Mini mental state examination; *p ≤ 0.05; **p ≤ 0.001.

### Sensitivity analysis

The analysis of the complete sample (n = 279) resulted in higher adjusted costs (+10,032 EUR; p = 0.1) and fewer adjusted QALY (−0.0074 QALY; p = 0.86) in the IG compared to the CG. The probability of cost-effectiveness at WTP thresholds of 0 EUR, 50,000 EUR and 250,000 EUR per QALY was 5%, 7% and 19%, respectively (Figure 2).

The use of the EQ VAS score resulted in fewer adjusted QALY (−0.0166 QALY; p = 0.71) in the IG compared to the CG. The probability of cost-effectiveness at WTP thresholds of 0 EUR, 50,000 EUR and 250,000 EUR per QALY was 7%, 7% and 22%, respectively (Figure 2).

The application of the German tariff for the EQ-5D index led to more adjusted QALY in the IG in comparison to the CG (+0.0034; p = 0.93) The probability of cost-effectiveness at WTP thresholds of 0 EUR, 50,000 EUR and 250,000 EUR per QALY was 7%, 13% and 37%, respectively (Figure 2).

## Discussion

The purpose of our study was to perform a cost-utility analysis of preventive home visits from a societal perspective in Germany. The results of our analysis showed that this preventive home visit programme is unlikely to be cost-effective. The probability of cost-effectiveness increased with higher WTP but remained low (39% at a threshold of 250,000 EUR per QALY). There was only a 7% chance that the preventive home visit programme is dominant (i.e. less costly and more effective) as indicated by the probability of cost-effectiveness at a WTP of 0 EUR per QALY. The sensitivity analysis showed that the results were robust to the way (German EQ-5D index, EQ VAS) QALY were calculated. The probability of cost-effectiveness never exceeded a probability of 40%.

Our interpretation of the results is based on the results of the CEAC. The common approach is the use of the ICER to interpret results. Yet, this approach is prone to bias. The ICER does not control for baseline differences between intervention and control group. Although in our study differences between groups at baseline were not statistically significant, even small difference may have huge impact on the point estimate of the ICER since costs and, in particular, QALY were very similar in both groups during follow-up.

After adjustment for baseline differences in potential prognostic variables, total costs during follow-up tended to be higher in the IG than in the CG, with costs for informal care and outpatient physician services being significantly increased. It is possible that the preventive home visits program has induced the participants’ demand for physician services and care provided by relatives and friends since the intervention explicitly aimed at identifying care deficits and motivating participants to seek solutions. Despite increased service use, the number of QALY was only marginally higher in the IG, resulting in low probability for cost-effectiveness.

There is one other cost-effectiveness analysis of preventive home visits in the literature which also used QALY as measure of health effects. Sahlen et al. reported that preventive home visits were cost saving or cost-effective dependent on the time horizon of the analysis and on the inclusion or exclusion of future health care costs in life years gained [11].

Two differences between our study and the study of Sahlen et al. should be considered. The first difference is the implementation of the intervention. Sahlen et al. performed two home visits per year over two years. In our study three visits in seven weeks were performed. It is possible that frequent visits over a longer time period are economically superior to a short term approach trying to give a stimulus for changes in behaviour and living environment. However, a study by Melis et al. showed that this is not necessarily the case [12]. This research group analysed a preventive home visit programme which performed a maximum of six visits over three months. The intervention was cost effective in terms of cost per successful treatment (prevented functional decline accompanied by improved mental well-being). The second difference is the age of the population and associated with this the choice of the effect measure. The sample of the study by Sahlen et al. had a mean age of 79.8 years [11] whereas the mean age in our sample was 84.8 years. We observed a decrease in quality of life between the baseline and the follow-up assessment in both groups which seems to be an age-related phenomenon. In the study by Sahlen et al. the HRQL measured with the EQ-5D index was higher (0.7) and assumed as stable over time.

There was only one statistically significant effect in our study. The number of deceased patients was lower in the IG than in the CG. In another analysis of their data, Sahlen et al. reported similar results [50]. They found that the risk of death decreased to 2.7% in the intervention group as compared to 4.8% in the control group. In our case the difference was larger (IG: 9% risk; CG: 18% risk) but this can be explained by the higher age of the population. Whether this effect is caused by the intervention should be evaluated by further research employing a longer time horizon and including more patients.

The main limiting factor of our study is the fact that there was only a non-significant reduction of nursing home admissions. Therefore the primary analysis failed to show effectiveness of the intervention. The reason for this is that a higher number of nursing home admissions was expected when planning the study. Eventually, a risk reduction of 45% was identified which was not statistically significant though. It is reasonable to assume that there is a chance to reach statistical significance for this relevant difference with a larger sample size. The added value of the present analysis is that we used a standard health economic measure of effectiveness (QALY) for which we found only a very small difference. By increasing the sample size to possibly reach statistical significance for the primary outcome the interpretation of the findings of our economic evaluation would probably not change substantially. The intervention would still be likely to have a pronounced and negative net-monetary benefit resulting from notable incremental costs and very small incremental QALY. This means that even if the intervention turned out to be effective based on the primary outcome it would not be likely to be cost-effective.

The following limitations should be considered in interpreting our findings. First of all, our sample may not be representative of the older population at high risk for admission to a nursing home in the two regions. We used a non-random sampling approach recruiting patients at baseline consecutively from GP practices, hospitals and the registration office in the two centres. However, 93% of people aged 70+ are regularly seen by a GP, which supports the representativeness of our population [51]. All consecutive patients were asked to participate in the study. However, some selection bias towards patients more willing and able to participate cannot be ruled out completely. Secondly, sample size might have been not sufficient enough to detect differences in outcomes. Sample size calculations were based on the number of prevented nursing home admissions, the primary outcome of the effectiveness study. Thirdly, we only observed our variables at baseline and one follow-up, reducing the ability to track more precisely health-related quality of life and costs over time. Therefore estimates in health status and costs more reflect the situation at 18 months rather than the health status and services used shortly after the intervention.

## Conclusion

The evaluated preventive home visits program is unlikely to be cost-effective. In the development of future programs aspects like the duration of the program, frequency of visits and the age of the target group should be considered.

## Abbreviations

CEAC: Cost-effectiveness-acceptability-curve
CG: Control group
EUR: Euro [€]
GDS: Geriatric depression scale
GP: General practitioner
HR: Hazard ratio
HRQL: Health related quality of life
IADL: Instrumental activities of daily living
ICER: Incremental cost-effectiveness ratio
IG: Intervention group
MMSE: Mini mental state examination
NMB: Net-monetary benefit
OLS: Ordinary least square
QALY: Quality-adjusted life year
RCT: Randomized controlled trial
RUD: Resource utilization in Dementia
SD: Standard deviation
UK: United Kingdom
VAS: Visual analogue scale
WTP: Willingness-to-pay
